# Crystal structures of IspF from *Plasmodium falciparum* and *Burkholderia cenocepacia*: comparisons inform antimicrobial drug target assessment

**DOI:** 10.1186/1472-6807-14-1

**Published:** 2014-01-10

**Authors:** Patrick EF O’Rourke, Justyna Kalinowska-Tłuścik, Paul K Fyfe, Alice Dawson, William N Hunter

**Affiliations:** 1 Division of Biological Chemistry and Drug Discovery, College of Life Sciences, University of Dundee, Dundee, DD1 5EH, UK; 2 Current address, Department of Crystal Chemistry and Crystal Physics, Faculty of Chemistry, Jagiellonian University, 30-060 Kraków, Poland

**Keywords:** Antimicrobial drug target, Isoprenoid biosynthesis, X-ray crystallography, Zn^2+^-dependent enzyme

## Abstract

**Background:**

2*C*-methyl-D-erythritol-2,4-cyclodiphosphate synthase (IspF) catalyzes the conversion of 4-diphosphocytidyl-2*C*-methyl-D-erythritol-2-phosphate to 2*C*-methyl-D-erythritol-2,4-cyclodiphosphate and cytidine monophosphate in production of isoprenoid-precursors *via* the methylerythritol phosphate biosynthetic pathway. IspF is found in the protozoan *Plasmodium falciparum,* a parasite that causes cerebral malaria, as well as in many Gram-negative bacteria such as *Burkholderia cenocepacia*. IspF represents a potential target for development of broad-spectrum antimicrobial drugs since it is proven or inferred as essential in these pathogens and absent from mammals. Structural studies of IspF from these two important yet distinct pathogens, and comparisons with orthologues have been carried out to generate reagents, to support and inform a structure-based approach to early stage drug discovery.

**Results:**

Efficient recombinant protein production and crystallization protocols were developed, and high-resolution crystal structures of IspF from *P. falciparum* (Emphasis/Emphasis>IspF) and *B. cenocepacia* (*Bc*IspF) in complex with cytidine nucleotides determined. Comparisons with orthologues, indicate a high degree of order and conservation in parts of the active site where Zn^2+^ is bound and where recognition of the cytidine moiety of substrate occurs. However, conformational flexibility is noted in that area of the active site responsible for binding the methylerythritol component of substrate. Unexpectedly, one structure of *Bc*IspF revealed two molecules of cytidine monophosphate in the active site, and another identified citrate coordinating to the catalytic Zn^2+^. In both cases interactions with ligands appear to help order a flexible loop at one side of the active site. Difficulties were encountered when attempting to derive complex structures with other ligands.

**Conclusions:**

High-resolution crystal structures of IspF from two important human pathogens have been obtained and compared to orthologues. The studies reveal new data on ligand binding, with citrate coordinating to the active site Zn^2+^ and when present in high concentrations cytidine monophosphate displays two binding modes in the active site. Ligand binding appears to order a part of the active site involved in substrate recognition. The high degree of structural conservation in and around the IspF active site suggests that any structural model might be suitable to support a program of structure-based drug discovery.

## Background

Isoprenoids contribute to a myriad of essential biological functions, for example as the prenyl groups that anchor proteins to membranes, as components of respiratory membranes, enzyme cofactors, and as scaffolds for the assembly of glycan structures [1-4]. Two distinct biosynthetic routes supply the universal isoprenoid-precursors isopentenyl pyrophosphate and dimethylallyl pyrophosphate. The mevalonate (MVA) pathway is present in animals, fungi and archaea while the 2*C*-methyl-D-erythritol-4-phosphate (MEP) pathway is widely distributed in bacteria, algae and several apicomplexan parasites, including *Plasmodium* species responsible for malaria [5,6]. Both MVA and MEP pathways occur in plants; the former operates in the cytoplasm and the latter in chloroplasts [7]. In most apicomplexan parasites the components of the MEP pathway are localized to an organelle known as the apicoplast, a relict chloroplast, which is also responsible for additional biosynthetic processes [8]. The organelle, which resembles a cyanobacterium is thought to have been acquired from an ancestral endosymbiotic event with an alga. All MEP pathway enzymes in *P. falciparum* are synthesized in the cytoplasm but they carry an apicoplast targeting sequence, which then drives localization [8]. The disruption of apicoplast replication results in parasites that are isopentenyl pyrophosphate auxotrophs [9].

**Figure 1:**
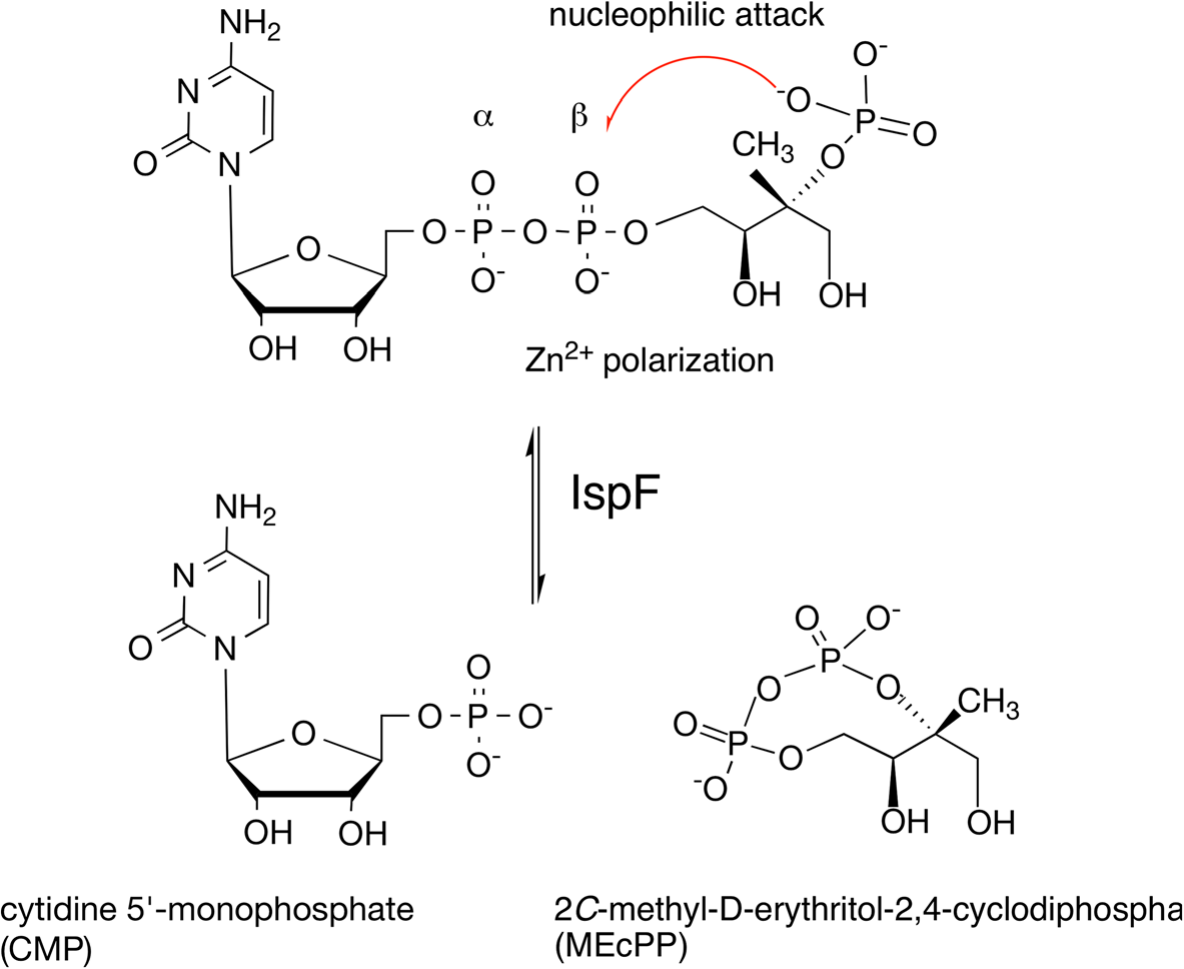
**Reaction catalyzed by IspF.** A catalytic Zn^2+^ aligns and polarizes the β-phosphate to support nucleophilic attack by the 2-phosphate to produce MEcPP and CMP.

Part of the interest in the MEP pathway concerns antimicrobial drug research and several observations support the idea that the pathway contains potential drug targets, enzymes whose inhibition may provide broad-spectrum antimicrobial activity [5,6]. We identified the Zn^2+^-dependent 2*C*-methyl-D-erythritol-2,4-cyclodiphosphate synthase [EC: 4.6.1.12] the fifth enzyme in the pathway as a target of interest. This enzyme, also known as IspF, catalyzes the conversion of 4-diphosphocytidyl-2*C*-methyl-D-erythritol-2-phosphate (CDP-MEP) to 2*C*-methyl-D-erythritol-2,4-cyclodiphosphate (MEcPP) and cytidine monophosphate (CMP, Figure 1). We considered a diverse set of criteria that have been established as key with respect to target assessment for early stage antimicrobial drug discovery [10]. These criteria include genetic and chemical validation of the target, and the availability of accurate structural information and appropriate reagents to guide the development of structure-activity relationships. Three primary factors were taken into consideration. First, genetic methods have identified that individual components of the pathway, including IspF are essential for bacterial survival. For example, the essentiality of the *ispF* gene for growth and survival of *Bacillus subtilis, Escherichia coli* and *Mycobacterium tuberculosis* is established [11-15]. Secondly, this pathway is absent from humans [5,6] potentially mitigating against toxicity issues. Thirdly, chemical validation of the pathway is provided by the antibacterial fosmidomycin, a potent inhibitor of 1-deoxy-D-xylulose 5-phosphate reductoisomerase, an enzyme that contributes to an early stage in the pathway. This last point regarding validation is reinforced by further work, which shows that fosmidomycin and derivatives are also efficacious in killing *P. falciparum*[16-18].

Previous studies have provided insight into aspects of IspF structure, specificity, and mechanism [19-22]. The structure of IspF has been derived from a dozen species in addition to the two new ones disclosed in this publication. More recently new IspF ligands including potent inhibitors have been identified [23-25], some of which have also been shown to inhibit the growth of *P. falciparum* within infected erythrocytes with respectable *IC*_50_ values of 1.4-1.6 μM [26]. These approaches have been complemented by NMR-based fragment screening and co-crystallization efforts targeting IspF from *B. pseudomallei* (*Bp*IspF) [27].

### Aims

We sought to further inform the assessment of IspF as a drug target with crystallographic studies of the enzyme from two serious human pathogens, the Gram-negative bacterium *Burkholderia cenocepacia* (*Bc*IspF) and the parasitic protozoan *Plasmodium falciparum* (*Pf*IspF).

## Results and discussion

### The structure of IspF

Efficient recombinant protein production systems, purification protocols and reproducible crystallization conditions have been established for *Bc*IspF and *Pf*IspF leading to yields of up to 25 mg L^-1^ and 2.5 mg L^-1^of *E. coli* culture respectively. High-resolution crystal structures of *Bc*IspF in complex with CMP or citrate, and of *Pf*IspF in complex with CDP, as well as an unliganded *Pf*IspF structure have been determined. Crystallographic statistics are presented in Tables 1 and 2.

**Table 1:** **Crystallographic statistics for *Pf *** **IspF**

**Structure/PDB**	***Pf *** **IspF:CDP/4C81**	**Unliganded *Pf *** **IspF/4C82**
Space group	*H*3	*H*3
Unit cell dimensions		
*a, b, c* (Å)	84.9, 84.9, 101.0	84.2, 84.2, 102.5
Resolution range^**a**^ (Å)	41.63-1.60 (1.69-1.60)	29.71-2.00 (2.11-2.00)
No. reflections	181781 (16968)	41802 (5780)
Unique reflections	35607 (5194)	18068 (2622)
Completeness (%)	99.4 (99.3)	98.6 (97.0)
*R*_merge_^**b**^ (%)	9.3 (42.0)	9.4 (48.2)
Multiplicity	5.1 (3.3)	2.3 (2.2)
< I/σ(I)>	11.1 (2.6)	5.3 (2.0)
Wilson *B* (Å^2^)	24.4	32.0
*R*_work_^**c**^/*R*_free_^**d**^ (%)	14.4, 16.5	19.4, 22.6
Number of residues/waters/ligands	156, 85 waters, 1 CDP, 2 Zn^2+^, 3 Cl^-^, 1 SO_4_^2-^	156, 94 waters, 2 Zn^2+^, 1 SO_4_^2-^
RMSD bond lengths (Å)/	0.005	0.019
Bond angles (º)	1.000	2.087
Mean *B*-factors (Å^2^)		
Overall	22.9	37.6
Main chain, side chain	25.1, 33.8	34.2, 39.5
Waters	35.2	47.8
Ligands	34.9, 28.3 (0.6, 0.4 CDP)	44.5 (Zn^2+^, SO_4_^2-^)
	35.8 (Zn^2+^, Cl^-^, SO_4_^2-^)	
Ramachandran plot		
Favoured regions (%)	98.6	95.3
Allowed regions (%)	0.7	4.0
Outliers	1 outlier: Asp79	1 outlier: Asp79

^a^Values in parentheses refer to the highest resolution shell. ^b^*R*_*merge*_ = ∑ _*hkl*_ ∑ _*i*_|I_*i*_(*hkl*) − < I(*hkl*) > |/∑_*hkl*_ ∑ _*i*_I_*i*_(*hkl*); where I_*i*_(*hkl*) is the intensity of the *i*th measurement of reflection *hkl* and < I(*hkl*) > is the mean value of I_*i*_(*hkl*) for all *i* measurements. ^c^*R*_*work*_ = ∑ _*hkl*_‖*F*_*o*_| ‒ |*F*_*c*_‖/∑|*F*_*o*_|, where *F*_*o*_ is the observed structure factor and *F*_*c*_ is the calculated structure factor. ^d^*R*_*free*_ is the same as *R*_*work*_ except calculated with a subset, 5%, of data that are excluded from the refinement calculations.

**Table 2:** **Crystallographic statistics for *Bc*** **IspF**

**Structure/PDB**	***Bc*** **IspF:CMP/4C8G**	***Bc*** **IspF:2CMP/4C8E**	***Bc*** **IspF:citrate/4C8I**
Space group	*C*2	*C*2	*C*2
Unit cell dimensions			
*a, b, c* (Å),	97.2, 88.6, 72.4,	97.9, 89.3, 73.0,	131.7, 52.5, 72.1,
β	103.9°	104.2°	94.6°
Resolution range (Å)	29.84-2.00 (2.10-2.00)	19.32-1.90 (2.00-1.90)	40.90-2.00 (2.10-2.00)
No. reflections	98451 (13335)	164120 (24760)	119715 (15957)
Unique reflections	38409 (5579)	47918 (6972)	33460 (4696)
Completeness (%)	96.0 (95.4)	99.9 (100.0)	99.4 (96.1)
*R*_merge_ (%)	11.0 (32.2)	13.8 (32.3)	3.2 (7.8)
Multiplicity	2.6 (2.4)	3.4 (3.6)	3.6 (3.4)
< I/σ(I)>	5.9 (2.7)	6.2 (3.1)	23.9 (12.8)
Wilson *B* (Å^2^)	16.5	16.3	18.7
*R*_work_/*R*_free_ (%)	23.7, 28.0	20.4, 25.1	15.6, 19.8
Number of residues	458, 173 waters,	483, 420 waters,	460, 320 waters,
Waters/ligands	3 CMP, 3 Zn^2+^, 1 Mg^2+^, 1 PO_4_^3-^	6 CMP, 3 Zn^2+^, 1 SO_4_^2-^	3 citrates,3 Zn^2+^, 1 PO_4_^3-^
		1di(hydroxyethyl)ether	
RMSD bond lengths (Å)/	0.020	0.011	0.020
bond angles (º)	2.198	1.526	2.101
Mean *B*-factors (Å^2^)			
Overall	14.5	17.2	18.9
Main chain, side chain	13.6, 15.3	18.7, 20.4	16.2, 20.1
Waters	16.5	29.9	26.7
Ligands	15.1 (CMP) 24.8 (Zn^2+^, Mg^2+^, PO_4_^3-^)	18.2 (CMP 1)	23.4, 36.8, 43.1 (citrates)
		28.7 (CMP 2)	26.1 (Zn^2+^, PO_4_^3-^)
		25.0 (other ligands)	
Ramachandran plot			
Favoured regions (%)	96.9	97.8	97.7
Allowed regions (%)	2.2	2.2	2.3
Outliers	4 outliers: Ser37A/C, Tyr29B, Gly17C		

**Figure 2:**
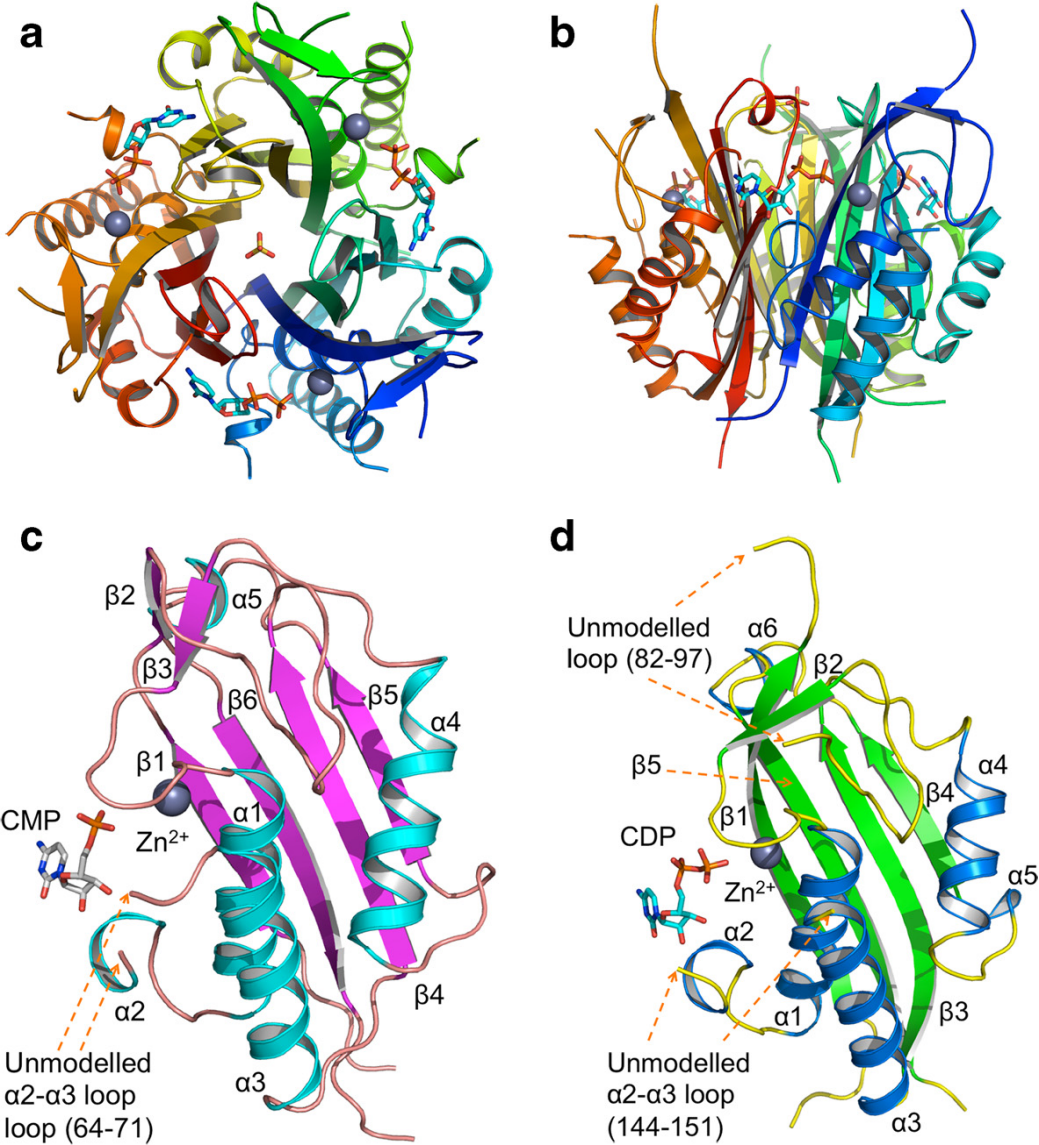
**Overall structure of IspF.** IspF forms a homotrimer. **a)** Axial view. **b)** Side view. The figure has been drawn using the coordinates for *Pf*IspF in complex with CDP; a trimer has been generated from symmetry-related subunits. Subunits (displayed as ribbons) are colored blue, orange and green respectively. Zn^2+^ cations are displayed as grey spheres, sulfate and CDP are displayed as sticks. The secondary structure of *Pf*IspF and *Bc*IspF subunits is shown in **c)** and **d)** respectively, similarly orientated. The location of missing loops is highlighted with arrows.

The calculated masses of the *Bc*IspF and *Pf*IspF subunits are 19.3 kDa and 20.5 kDa respectively and the polypeptide folds into a single α/β domain that consists of a four-stranded mixed β-sheet on one side with three α-helices on the other (Figure 2). IspF forms a stable homotrimer as observed in size-exclusion chromatography, which returns an estimate of 60 kDa for each. In all IspF crystal structures three subunits form a compact bell-shaped assembly approximately 45 Å by 60 Å in the axial and equatorial dimensions respectively (Figure 2). A surface area approximately equivalent to that of one subunit is buried on oligomerization. The accessible surface area (ASA) of an IspF monomer averages out as approximately 8130 Å^2^ for *Bc*IspF and 8680 Å^2^ for *Pf*IspF. The ASA for the *Bc*IspF trimer is 16080 Å^2^, and for the *Pf*IspF trimer 18180 Å^2^. In the case of *Bc*IspF the asymmetric unit consists of three subunits, chains A, B, C, which are related by three-fold non-crystallographic symmetry (NCS). NCS is high with least squares overlays of 161Cα positions in the range 0.2 - 0.3 Å. One subunit is present in the asymmetric unit of *Pf*IspF and the trimer is generated by the symmetry operations (−x + y, -x, z) and (−y, x-y, z). A least squares superimposition of Cα positions from both *Pf*IspF structures matches all 156 residues with an RMSD of 0.3 Å. Since there are no pronounced differences between the CDP bound and unliganded *Pf*IspF structures only the former is discussed.

Structural comparisons of IspF orthologues from the Protein Data Bank (PDB) were carried out using the *DALI* server [28]. Pairwise sequence identities range from 28 to 90%, Z scores from 18 to 31 and RMSD values from 0.3 to 2.5 Å. The apicoplast targeting sequence of *Plasmodium* spp. and the chloroplast targeting sequence of *Arabidopsis thaliana* IspF were excluded in the calculation of sequence identities. Using representative IspF structures from each of the twelve unique organisms in the PDB, an average RMSD of Cα positions was calculated against both proteins. The mean of the average RMSD was 1.5 Å and therefore the comparisons are indicative of an enzyme that has a highly conserved structure.

**Figure 3:**
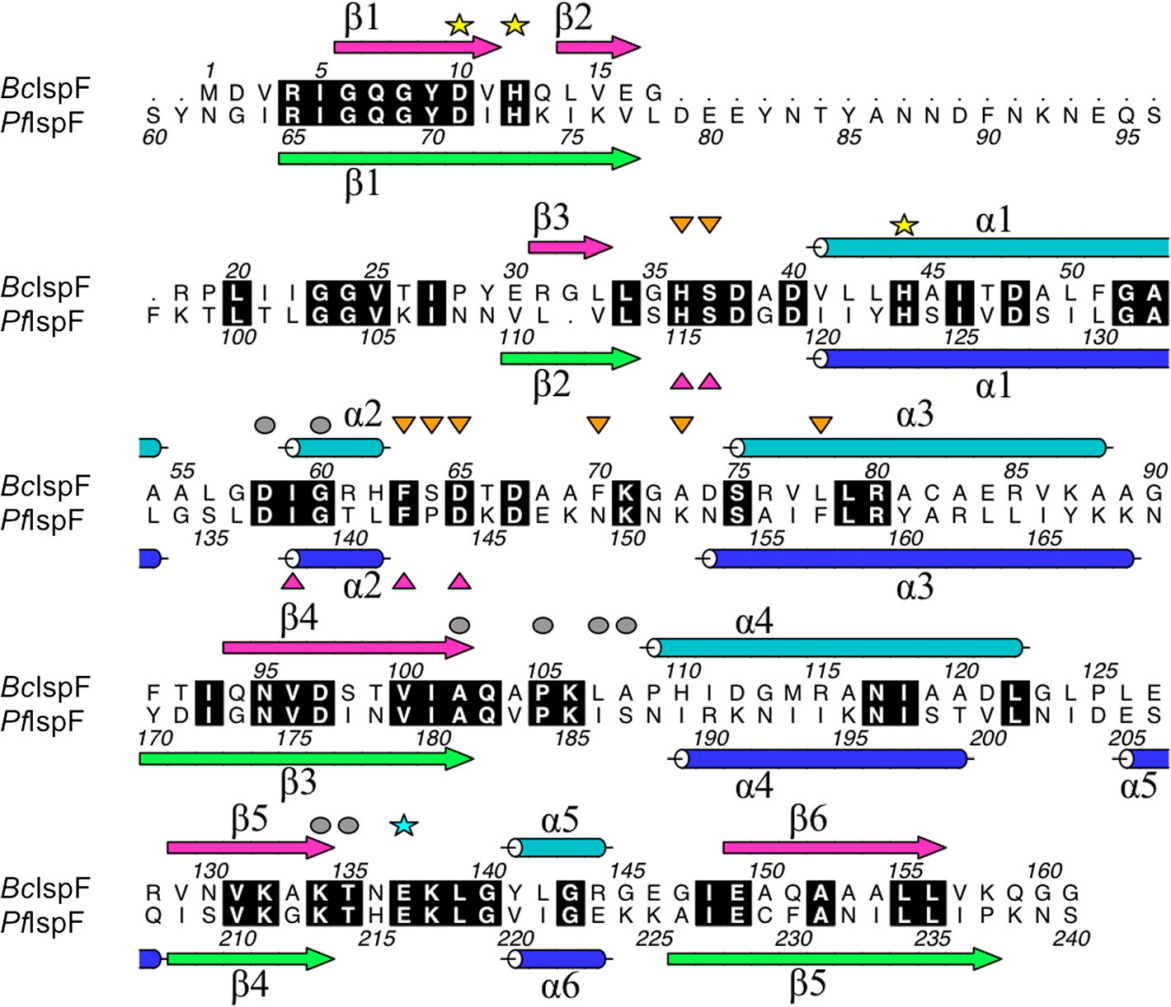
**Structure-based sequence alignment of *Bc*IspF and *Pf*IspF.** The alignment has been annotated with elements of secondary structure. Strictly conserved residues are highlighted in black. Three residues that coordinate Zn^2+^ are marked with a yellow star. Residues that bind CDP or CMP 1, are marked with a grey disk and those that bind CMP 2 with an orange triangle. Residues that bind MEcPP in *Ec*IspF [20] are marked with a magenta triangle. A conserved glutamate that binds either Mg^2+^ or Mn^2+^ in *Ec*IspF structures is highlighted with a cyan star. This figure was prepared using *ALINE*[29].

The two IspF variants studied here, with sequence identity of 38%, a DALI Z score 23 and RMSD value typically around 1.7 Å, can be taken to represent a comparison of structures that are amongst the most divergent. A structure-based sequence alignment of *Bc*IspF with *Pf*IspF is shown in Figure 3 and this, together with comparisons based on consideration of 854 IspF sequences, provides a context in which to discuss key features. *Pf*IspF contains a 19 residue insertion at the N-terminus, Lys76 - Glu94, immediately after β1 that does not align with bacterial IspF sequences. Here, residues Tyr82 - Phe97 could not be modeled due to the lack of ordered electron density (Figure 2c).

The most similar structures of each new IspF structure were noted. *Bc*IspF aligns 151 residues with an RMSD of 0.4 Å to *Bp*IspF (*Z* = 31, 90% sequence identity). *Pf*IspF aligns 143Cα positions with an RMSD of 0.8 Å, and 151 residues with an RMSD of 1.2 Å with subunits of the enzyme from *P. vivax* (*Pv*IspF, Z = 25, 66% sequence identity) and *A. thaliana* (*At*IspF, Z = 25, 42% sequence identity) respectively. The *Pv*IspF structure at 2.3 Å resolution was deposited in the PDB without an associated publication [PDB: 3B6N]. Although Zn^2+^ has been modeled at the active site (discussed below), the corresponding *B*-factor of 119 Å^2^ is 2.6 times greater than the average *B*-factor of the protein atoms, 46 Å^2^, and the side chain of a coordinating histidine, His170, is poorly oriented. We conclude that a water molecule may be a more likely occupant of this metal binding site. In contrast, in our structure of a *Plasmodium* IspF, which is at 1.60 Å resolution, the *B*-value for Zn^2+^ of 20 Å^2^ is lower than the average *B*-factor over all the protein atoms of 29 Å^2^.

**Figure 4:**
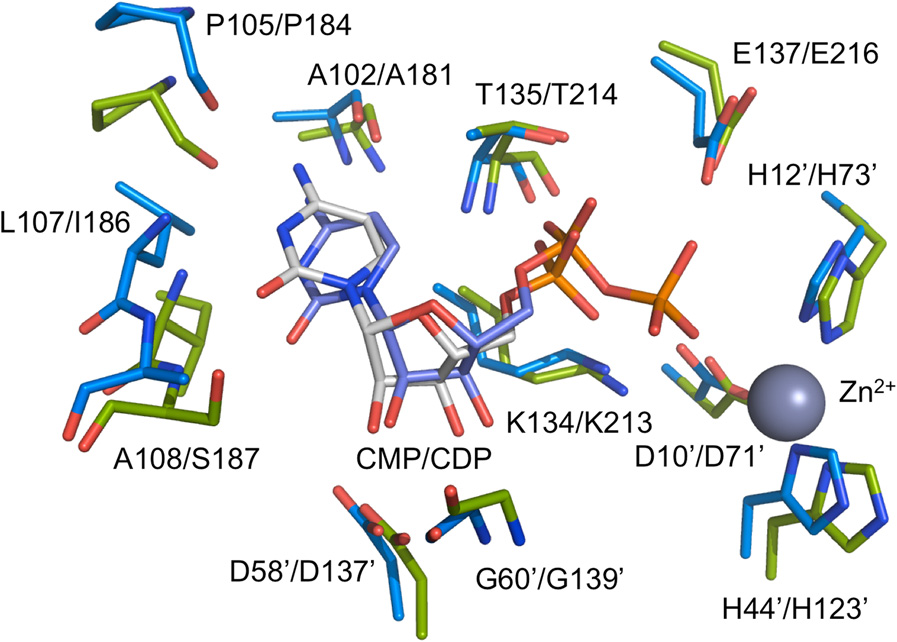
**Superposition of the active site in *Bc*IspF and *Pf*IspF.** Residues that bind CMP or CDP are displayed as sticks. Sequence numbers for *Bc*IspF are followed by those for *Pf*IspF, and residues contributed from the adjacent subunit are marked with’. *Bc*IspF and *Pf*IspF are colored cyan and green respectively. Key interactions are detailed in the text.

The enzyme active site is located in a cleft at the interface of two subunits (Figure 2a) and here Zn^2+^ is coordinated by the side chains of two histidines and an aspartate [19]. The coordinating residues are Asp10, His12 and His44 in *Bc*IspF, Asp71, His73 and His123 in *Pf*IspF (Figures 3 and 4). These residues are strictly conserved in IspF. In the *E. coli* enzyme (*Ec*IspF) Glu135, helps coordinate either a Mg^2+^ or Mn^2+^ together with the bridging phosphates of the substrate or CDP [19,20]. Although the residue is conserved as Glu137/216 (Figure 4) we do not observe any cation binding here. In 81% of IspF sequences a glutamate is observed at this position, and this is conservatively replaced by aspartate in a further 18%.

The highly conserved histidine and serine combination (*Pf*IspF: His115-Ser116, *Bc*IspF: His36-Ser37, identical in over 96% IspF sequences) located on the loop that leads to α1, adjacent to the Zn^2+^ binding site (Figure 2c, d), display elevated *B*-factors in all structures and in one chain of the *Bc*IspF:CMP complex the electron density was of insufficient quality to allow the region to be modelled, indicating a degree of flexibility. Electron density is absent or poorly defined for the loop linking α2 to α3 in many of the IspF structures. This segment occurs at one side of the active site (Asp144 - Asn151 in *Pf*IspF) and (Phe63 - Ala73 in *Bc*IspF). This α2 - α3 loop could be modeled in the *Bc*IspF:2CMP complex although the residues have elevated *B*-factors (average 47 Å^2^), compared to the average *B*-value for the entire protein (19 Å^2^). The same loop was also modelled for chain A in the *Bc*IspF-citrate complex. These observations are consistent with the inference that the α2 - α3 loop on one side of the active site is flexible [16,17].

A hydrophobic cavity occurs at the core of the IspF trimer. In *Bc*IspF the cavity is lined by the side chains of Tyr9, Val11, Ile101 and Tyr141, and the floor of the cavity is formed by a ring of six hydrophobic residues, Val3 and Leu155 donated by all three subunits. Similarly in *Pf*IspF side chains of Tyr70, Ile72, Ile180 and Val220 line this cavity, and the side chains of Phe230 form the floor of the cavity. Anions such as sulfate, phosphate or pyrophosphate bind at the entrance to this cavity in several IspF structures, for example phosphate is present in *At*IspF [30]. In the structure of *Bc*IspF crystallized with (NH_4_)_2_SO_4_ as a precipitant, a sulfate is bound by the side chain of Arg144. In *Pf*IspF, a sulfate binds close to this site but dislodged such that anion coordination involves the main chain amides of nearby residues, Val220 from all three subunits, rather than a basic side chain. When *Bc*IspF is crystallized in the presence of Na_2_HPO_4_, Tyr141 amide groups bind a phosphate in a similar fashion. Isoprenoid-species such as geranyl- or farnesyl-pyrophosphate have been observed in the central cavity of some IspF trimers and a possible role in feedback regulation has been suggested [31-33]. A molecule of di(hydroxyethyl)ether; a likely decomposition product or impurity of PEG 3350 used in crystallization, occupies the cavity in the *Bc*IspF:2CMP structure (Table 2), possibly mimicking a prenyl chain. A solvated Mg^2+^ with octahedral coordination is present in the cavity of the *Bc*IspF:CMP structure forming interactions similar to those observed in the structure of IspF from *B. pseudomallei*[27], data not shown.

### Complexes with CMP and CDP

Residues from both subunits interact with and position cytidine nucleotides in the active site. These compounds constitute fragments of the substrate and provide clues about aspects of molecular recognition. The structure-based sequence alignment (Figure 3) and overlay of the active sites of *Bc*IspF:CMP and *Pf*IspF:CDP complexes (Figure 4) indicate that the key enzyme-ligand interactions and aspects of the structure that are implicated in substrate specificity and the mechanism are highly conserved. Note that in the *Pf*IspF:CDP complex two conformers of CDP are present that differ in the orientation of the β-phosphate group. These refined satisfactorily at occupancy levels of 0.6 and 0.4.

In *Bc*IspF, carbonyl groups from Ala102 and *cis*-Pro105 accept hydrogen bonds donated by cytosine N4, and the amide groups of Leu107 and Ala108 donate hydrogen bonds to cytosine N3 and O2 respectively. The ribose O2’ forms a hydrogen bond with Asp58 from the adjacent subunit while the amide of Gly60, also from the adjacent subunit, hydrogen bonds to the ribose O3’. Thr135 amide and side chain hydroxyl groups form hydrogen bonds to the α-phosphate. Lys134 NZ interacts with the CMP α-phosphate, and may contribute to catalysis by helping to position the transition-state intermediate or, in conjunction with Zn^2+^, to align the substrate for nucleophilic attack. This interaction in *Bc*IspF is reminiscent of that previously reported for the active site Lys132 in *Thermus thermophilus* IspF [34], except that in that case the lysine interacts with the β-phosphate. The side chain of Lys106 is positioned over the cytosine and contributes van der Waals contacts to stabilize the ligand pose. For clarity this residue is omitted in Figure 4.

We analysed the levels of conservation of these eight IspF residues detailed as interacting with CMP or CDP. The residues for which the side chain is key, the glycine and *cis*-proline are highly conserved in IspF, at greater than 95%. Where main chain functional groups are used the nature of the side chain is less important and the level of conservation drops to 37% for the equivalent of Leu107 to 62% for Ala102 in *Bc*IspF. Lys134 is anomalous. This is only lysine in 20% of the sequences and is more often a threonine, 76% of sequences. Presumably the reduction in size allows space that a water molecule could occupy to help facilitate substrate binding.

**Figure 5:**
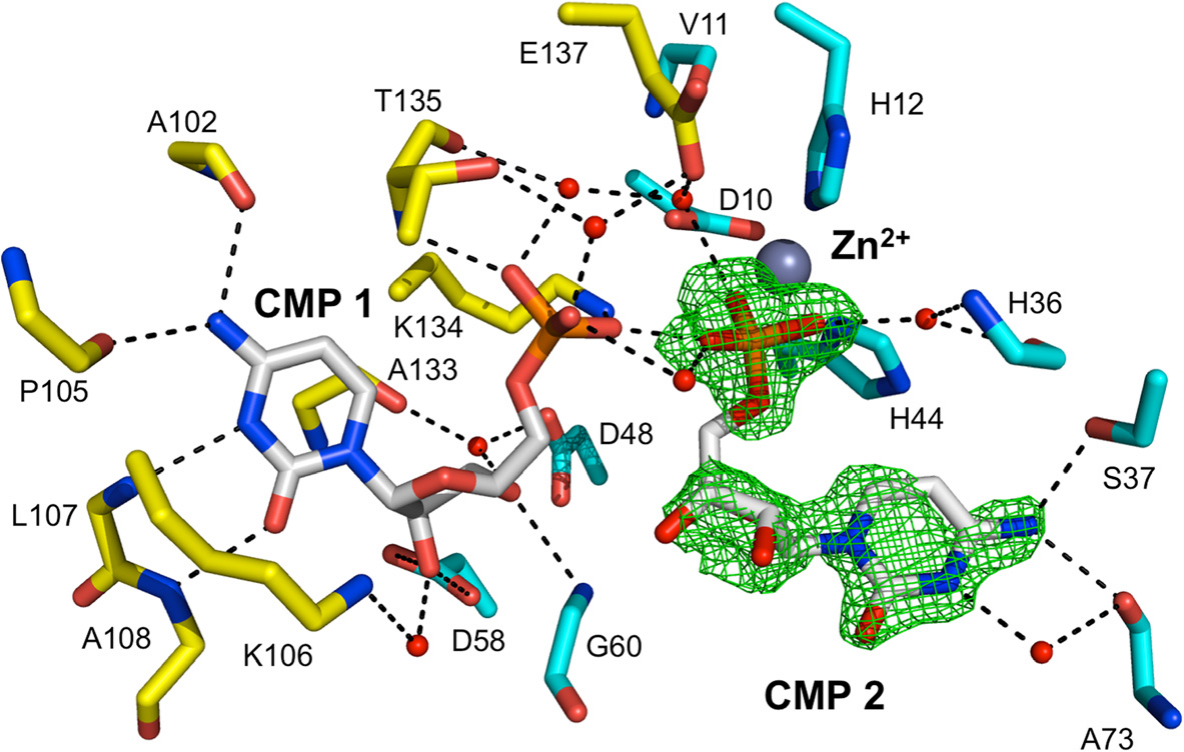
**Binding pose of two cytidine nucleotides in *Bc*IspF.** Hydrogen bonding interactions between *Bc*IspF, water molecules and both CMP molecules in one active site are depicted with black dashed lines. Residues that contribute to binding interactions only *via* the main chain are depicted without side chain atoms. Solvent molecules are depicted as red spheres. Components of one subunit are shown with C atoms yellow and for the other subunit, cyan. Electron density is displayed for CMP 2 as green chicken-wire corresponding to an |F_o_-F_c_| map (omit map) contoured at 2.5 σ.

**Figure 6:**
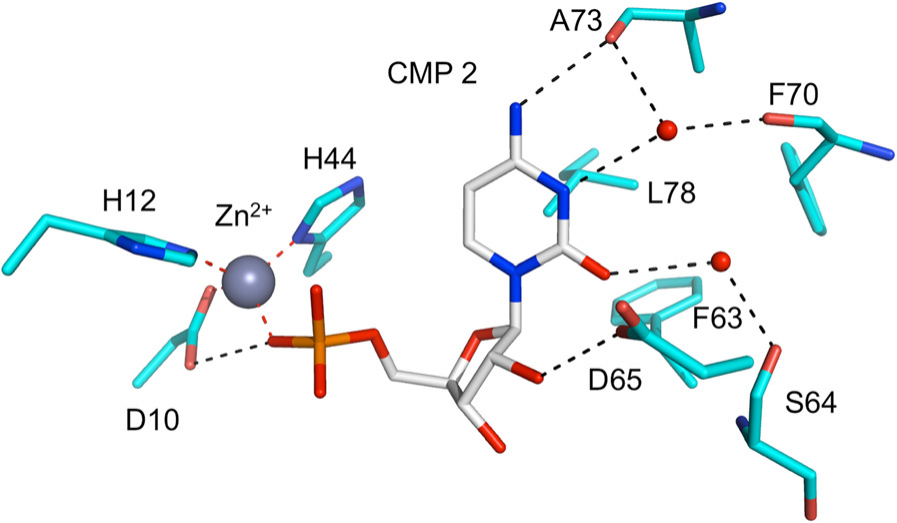
**Key residues and interactions to bind CMP 2 in *Bc*IspF.** The color scheme is the same as used in Figure 5.

**Figure 7:**
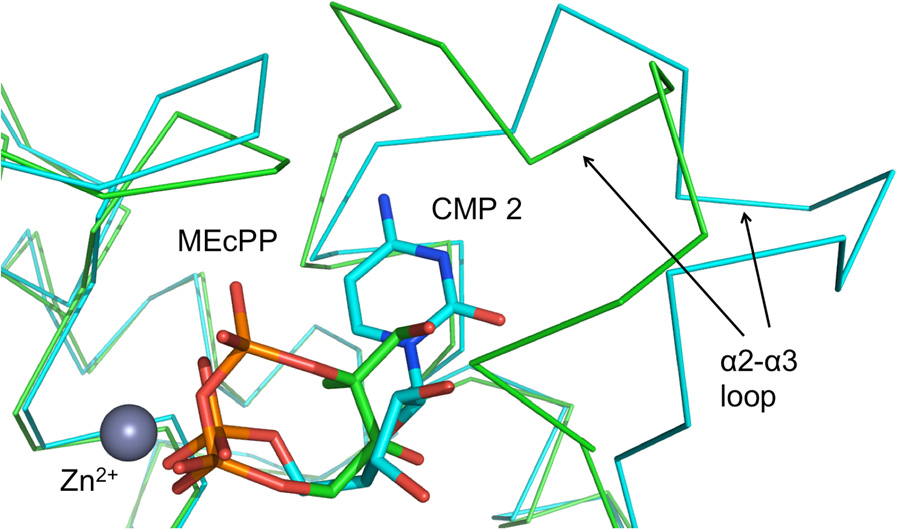
**CMP 2 occupies the same site as MEcPP.** An overlay of the *Ec*IspF product complex and *Bc*IspF:CMP 2. CMP 2 binds in a similar site as MEcPP and also coordinates Zn^2+^. The α2-α3 loop at one side of the binding site (*Ec*IspF: Phe61-Ser73; *Bc*IspF: Phe63-Ser75) adopts a different conformation.

Surprisingly*,* a second molecule of CMP was observed in the active site when *Bc*IspF was co-crystallized in the presence of 10 mM ligand. This second CMP is likely due to the high concentration used and represents an artifact of crystallization, unlikely to have physiological significance. This new cytidine-binding site is referred to as position 2 and residues that bind this ligand are from the same subunit that binds the active site Zn^2+^, indeed the CMP 2 phosphate coordinates the metal ion (Figure 5). The binding of CMP 2 within the three active sites of the asymmetric unit is very similar, for example the main chain conformation of the α2-α3 loop and the positions of Leu78 and His36 interacting with van der Waals forces on either side of the pyrimidine and helping to position it is essentially the same. In addition the position of Phe63 that helps to position Leu78 is also conserved. Phe63 is strictly conserved in 96% of IspF sequences, Leu78 in 77%. However, there are differences in the orientation of two side chains that change the detail of the hydrogen bonding interactions with CMP 2. In active site A (Figure 6) two solvent mediated links are noted involving the carbonyl groups of Ser64, Phe70 and Ala73 and cytosine O2 and N3. A direct hydrogen bond is formed between N4 and the Ala73 carbonyl. The Phe70 and Ala73 carbonyl groups position a water molecule that binds cytosine N3 and the Ala73 carbonyl also interacts directly with cytosine N4. The carboxylate of Asp65 binds to the ribose O2’. In the other active sites (not shown), different rotamers of Ser37 place the side chain OG to accept a hydrogen bond from N4 but for Asp65 the rotamer orientations results in functional groups too distant for an interaction with the ribose. Overlay with the *Ec*IspF-product complex shows that CMP 2 binds in the same location as MEcPP [20] and indeed exploits interactions with the same residues, the majority of which are conserved (Figures 3 and 7). Specifically, in common with observations regarding recognition of the pyrimidine moiety of substrates we note that residues that use side chain functional groups to interact with ligands in the MEcPP binding site are well conserved, those that use main chain groups less so. Ser37 is strictly conserved and Asp65 occurs in about 70% of IspF sequences but Ser64, Phe70 and Ala73 are strictly conserved in 4%, 43% and 58% respectively. The latter two residues are not conserved in *Pf*IspF, being replaced by asparagine and lysine respectively (Figure 3).

The binding pose of CMP 2 offers clues about molecular features that might be considered for ligand design targeting this area of the active site. Potential ligands could, for example, aim to extend out to recreate the hydrogen bonding interactions displayed by the two ordered water molecules shown in Figure 6. It might also be useful to test placement of a basic component to interact with the acidic Asp65.

### *Bc*IspF-citrate complex

**Figure 8:**
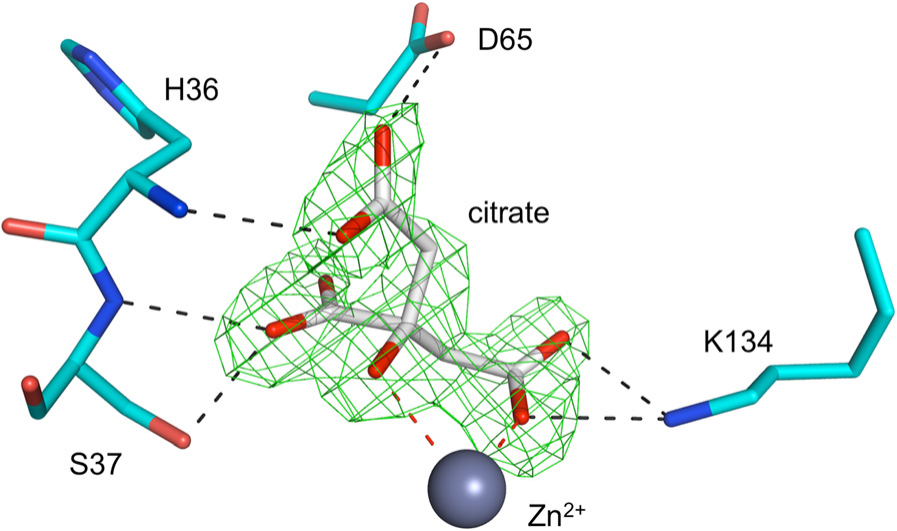
**Citrate binding to Zn**^**2+ **^**in *Bc*IspF.** A well-resolved citrate binds to Zn^2+^. An omit map is displayed for citrate in green chicken wire contoured at 3 σ. Residues are shown as sticks and a grey sphere represents Zn^2+^. Interactions are marked with dashed lines. Solvent mediated interactions are not shown.

Attempts to produce unliganded *Bc*IspF crystals for ligand or fragment soaking experiments initially appeared to be successful and highly ordered samples were obtained. However, citrate was present in the best crystallization conditions and the electron density maps were consistent with this molecule coordinating the metal ion and therefore complicating ligand-binding studies. Two binding modes were observed. Citrate was well defined in one active site with an average *B*-factor of about 23 Å^2^, but less so in the other two where it was modeled with occupancy 0.75 and the average *B*-factors are approximately 37 and 43 Å^2^. For comparison, the average *B*-factor for protein atoms in this structure is approximately 19 Å^2^. The best-defined citrate (Figure 8) binds to the active site Zn^2+^*via* both carboxylate and hydroxyl groups and this is also observed at one other site. Here the citrate forms hydrogen-bonding interactions with the main chain amides of His36 and Ser37, and side chains of Asp65 and Lys134. The interaction involving Asp65 is likely a consequence of the low pH at which crystals were obtained. Additional solvent mediated interactions to link citrate to the protein also occur (not shown). The normally flexible and poorly ordered α2-α3 loop appears to lock down over the bound citrate adopting a configuration similar to that observed in the *Ec*IspF-MEcPP complex [20]. In the other citrate-binding pose (not shown) only a carboxylate coordinates to Zn^2+^ but hydrogen bonds are also formed exploiting the same functional groups on the protein, the amides of His36 and Ser37, Ser37 OG, and Lys134 NZ. It is clear from these results, that any attempts to investigate ligand binding at the Zn^2+^ site will require crystallization conditions that do not include citrate.

### Co-crystallization with potential Zn^2+^ coordinating ligands

A screen of Zn^2+^-binding ligands previously identified L-tryptophan hydroxamate as a ligand for IspF suggesting that the presence of such a group may anchor ligands in the active site [25]. Subsequent studies with *Bp*IspF reported a number of metal ion coordinating ligands [27]. Crystallization trials of *Pf*IspF and *Bc*IspF in the presence of several such Zn^2+^ coordinating ligands (e.g. fosmidomycin, benzohydroxamic acid, acetohydroxamic acid and pyrithione), testing a range of concentrations together with the absence or presence of CMP were carried out. Citrate was not present in these crystallization trials. Numerous datasets have been measured and analyzed but we have not observed any evidence of selected ligands at the Zn^2+^ site. In one case a 2.0 Å resolution dataset was obtained from *Bc*IspF co-crystallized with 10 mM pyrithione. It quickly became evident that neither Zn^2+^ nor pyrithione were present in the structure (not shown) and this suggests that the ligand may have extracted Zn^2+^ from the active site.

## Conclusions

The structures of IspF from a bacterial (*Bc*IspF) and a protozoan (*Pf*IspF) pathogen have been determined and compared to orthologues. A high degree of structural similarity in and around the active sites is noted. These proteins have been assessed for suitability as platforms for structure-based drug discovery. The cytidine-binding pocket is relatively rigid in IspF, whereas the pocket that binds the methylerythritol-phosphate portion of the substrate is relatively flexible. Numerous attempts to obtain the structure of protein-ligand complexes failed suggesting that the crystallization conditions may need to be adjusted or alternatives found. We note that in each case high ionic strength was necessary to obtain well-ordered crystals. Successful co-crystallization with small molecule ligands has been reported for *Ec*IspF and *Bp*IspF [24,25,27] and given the high degree of structural similarity in the IspF active site it might be prudent to use those enzymes for early stage drug discovery studies. Previously [26], *At*IspF was used as a surrogate for *Pf*IspF due to difficulties in working with the later. We also noted problems with the solubility of *Pf*IspF and subsequently identified conditions to circumvent this issue. However, given the high level of conservation we have described then any IspF might be considered a suitable surrogate for the apicomplexan enzymes. The unexpected observations that a second molecule of CMP can occupy the active site as well as the presence of citrate interacting with Zn^2+^ in *Bc*IspF are noteworthy. These ligands provide templates for ligand design addressing metal ion coordination and interactions in the MEcPP-binding pocket.

## Methods

### Protein production

The gene encoding *Bc*IspF was amplified from *B. cenocepacia* genomic DNA (strain J2315) by PCR using 5′- CAT ATG GAC TTC AGA ATC GGA CAA GG −3′ and 5′- GGA CCT CAG CCG CCC TGC TTC ACC −3′ as the forward and reverse primers respectively (Thermo Scientific). The PCR product was ligated into TOPO-Blunt-II (Invitrogen) then subcloned into a modified pET15b vector (Novagen), which produces a histidine-tagged protein with a Tobacco Etch Virus (TEV) protease site. A single nucleotide error in the forward primer gave a single amino acid mutation F3V. The gene encoding IspF from *P. falciparum* strain 3D7, [GenBank:XP_001349603], was synthesized (Genscript) with codons optimized for recombinant expression in *E. coli.* The sequence encoding residues 1–59, a predicted apicoplast targeting sequence, was excluded and the codon for Cys60 was replaced with one for serine. This synthetic gene was sub-cloned into the same modified pET15b vector as *Bc ispF*. Experimental procedures for recombinant protein production using *E. coli* BL-21 (DE3) Gold following published methods [19].

### Purification of *Pf*IspF

*Pf*IspF precipitates at temperatures below 18°C therefore all procedures were performed at room temperature unless otherwise stated. Cells were harvested by centrifugation at 3000 *g* for 30 minutes at 18°C and re-suspended in 100 mM KCl, 100 mM L-arginine, 50 mM CHES, pH 9.5 (buffer A). DNaseI and a protease inhibitor tablet (EDTA-free, Roche) were added prior to cell lysis by passage through a French pressure cell at 16000 *psi*. Cell lysate was clarified by ultracentrifugation at 40000 *g* for 30 minutes at 18°C (Avanti centrifuge, Beckmann) and the supernatant was syringe filtered (Sartorius).

Lysate was loaded onto a nickel ion affinity chromatography column (5 mL HisTrap HP, GE Healthcare) using an FPLC system (Äkta Explorer, GE Healthcare). A linear concentration gradient of imidazole was applied with elution of *Pf*IspF occurring at 200 mM mM imidazole. The fractions containing IspF were pooled and dialyzed against buffer A. The affinity tag (His-tag) was cleaved with His-tagged TEV protease at 20°C for 16 hours. Protease-treated sample was re-applied to a nickel ion affinity chromatography column, and the cleaved protein eluted. Fractions were analyzed by sodium dodecyl sulfate polyacrylamide gel electrophoresis (SDS-PAGE) and those containing *Pf*IspF were pooled. The sample was further purified on a size-exclusion chromatography column (Superdex 200 26/60, GE Healthcare) equilibrated with buffer A. This column had been calibrated with molecular weight standards (Bio-Rad). The level of *Pf*IspF purity was confirmed by SDS-PAGE and matrix-assisted laser desorption/ionization time-of-flight mass spectrometry. Protein concentration was estimated spectrophotometrically using a theoretical extinction coefficient, ϵ: 11920 M^-1^ cm^-1^ at 280 nm, calculated using *ProtParam*[35].

### Purification of *Bc*IspF

*Bc*IspF was purified in similar fashion to *Pf*IspF except that a different buffer, (100 mM NaCl, 100 mM Tris–HCl, pH 7.5, buffer B) was used. Removal of the His-tag reduced the solubility of the protein significantly. Once this problem was identified the tag was left in place. The temperature sensitivity observed for *Pf*IspF was not seen for *Bc*IspF and samples were kept either on ice or at 4°C throughout. *Bc*IspF concentration was measured using the Bradford assay (Thermo Scientific).

### Screening for crystallization conditions

An automated liquid-handling system (Phoenix, Art Robbins) was used for screening crystallization conditions in combination with commercially available screens. All samples were filtered through a 0.1 μm PVDF filter (Ultrafree, Millipore) immediately prior to screening. Sitting-drops were formed by mixing 100 nL sample with either 100 or 200 nL of reservoir. Reservoir volumes were 60 μl. Conditions identified from the screens were subsequently scaled up and optimized.

### Crystallization of a *Pf*IspF:CDP complex and unliganded *Pf*IspF

*Pf*IspF was concentrated to 6 mg mL^-1^ using a 10 kDa cutoff centrifugal filter (Vivaspin 20, Sartorius) to provide a stock solution for crystallization. The sample was supplemented with MgCl_2_ (2 mM) and CDP disodium salt (2 mM, Sigma-Aldrich). Hanging drops were prepared consisting of 2 μl sample mixed with 2 μl of reservoir (1.8-2.5 M (NH_4_)_2_SO_4_, 5 mM ZnCl_2_ and 100 mM Bis-Tris, pH 5.5). Diffusion against 1000 μl of reservoir led to the formation of small prismatic crystals (approximate dimensions 0.15 × 0.15 × 0.15 mm) within 2–4 days. Isomorphous crystals were also obtained with 10 mM fosmidomycin present in the crystallization drop. Crystals were transferred to drops containing saturated sucrose solution, for ~5 seconds before flash cooling in liquid nitrogen.

### Crystallization of *Bc*IspF complexes

*Bc*IspF was concentrated to 5 mg mL^-1^ in buffer B, similar to *Pf*IspF and the solution adjusted to include 2 mM MgCl_2_ and 20 mM CMP disodium salt (Sigma-Aldrich). A second sample was prepared at 10 mg mL^-1^ in buffer C (100 mM NaCl, 2 mM MgCl_2_, 100 mM sodium formate, pH 5.0) to which CMP (2 mM) was also added.

The *Bc*IspF CMP 2 structure was obtained from crystals grown by mixing 2 μL of the sample in buffer B with reservoir (2 μL) containing 25% w/v PEG 3350, 2.2 M (NH_4_)_2_SO_4_, 0.1 M sodium formate, pH 5.0, and 1% v/v dioxane. Orthorhombic blocks reached maximum size within ten days. A crystal of approximate dimensions 0.15 × 0.10 × 0.10 mm was used for data collection. Buffer B supplemented with 20% v/v glycerol was used as a cryoprotectant.

The second sample gave the *Bc*IspF CMP structure. This was crystallized by mixing 2 μL with 4 μL reservoir containing 5% w/v PEG 1000, 33% v/v ethanol, 0.1 M Na_2_HPO_4_, pH 4.0, and 2% v/v dioxane. A crystal of approximate dimensions 0.25 × 0.10 × 0.10 mm grew over six days and was used for data collection. A mixture of buffer C with reservoir (1:2), replacing PEG 1000 with PEG 4000 at 25% w/v, was used as a cryoprotectant.

The *Bc*IspF:citrate structure was derived from protein concentrated to 10 mg mL^-1^ in buffer D (100 mM NaCl, 100 mM sodium formate, pH 5.0), and crystallized by mixing (1.5 μL) with a reservoir (3 μL) containing 5% w/v PEG 1000, 36% v/v ethanol, 0.1 M Na_2_HPO_4_ and 0.1 M citric acid, pH 4.2. A different crystal form to that previously found, grew within 4 days to approximate dimensions 0.4 × 0.1 × 0.1 mm. A mixture of sample buffer with reservoir (1:2), replacing PEG 1000 with PEG 4000 at 25% w/v, was used as a cryoprotectant. Datasets for the *Bc*IspF complex crystals were collected on the in-house X-ray system as described for *Pf*IspF.

Crystals were screened in-house using a rotating-anode X-ray source (MicroMax 007 HF, Rigaku) equipped with a dual image plate detector (RAXIS IV^++^). Diffraction data from the *Pf*IspF:CDP complex were collected on beamline ID29 at the European Synchrotron Radiation Facility (ESRF, Grenoble) at −170°C using an ADSC Quantum 315r CCD detector. Data from the crystals grown in the presence of fosmidomycin were collected in-house. Subsequently it was determined that fosmidomycin was not ordered and this is therefore considered to be an unliganded *Pf*IspF structure. Datasets for the *Bc*IspF complex crystals were all collected on the in-house X-ray system described above.

### Data processing, structure solution and refinement

Diffraction data were indexed and integrated using *MOSFLM*[36] or *XDS*[37] and scaled with *SCALA*[38] with 5% of data flagged for *R*_free_ calculations. The *R*_free_ flag was maintained for isomorphous structures. Initial structures were solved by molecular replacement using *PHASER*[39]. Different search models, edited to remove all non-protein atoms, were used for these calculations. The coordinates of *E. coli* IspF [PDB:1GX1] with ~33% sequence identity, [19] and *B. pseudomallei* [PDB:3F0G] 63% identity, [27] were used to determine the *Pf*IspF:CDP and *Bc*IspF:2CMP structures respectively. *B*-factors of the replacement models were adjusted to match the Wilson *B-*factor derived from scaling of the data. Subsequently these parent structures were used as the starting points for analyses of the other structures. Crystals of *Pf*IspF display differing degrees of twinning as recognised in *SCALA* and a twin fraction of 0.14 was applied to the CDP complex.

Refinement calculations were performed with *REFMAC5*[40] and the resulting models together with electron density and difference density maps were inspected in *COOT,* which also allowed for model adjustment to improve the fit to density [41]. Water molecules, ions, ligands and conformational rotamers were added to the models as the refinements progressed. In the case of citrate molecules binding to *Bc*IspF, one site was well ordered but the other two sites displayed more diffuse electron density and a decision was taken to set the occupancy to 0.75. These then refined satisfactorily with no residual features in the resulting difference density maps. Strict NCS restraints were employed in the early stages of refinement and then removed towards the latter stages. To confirm the presence of ligand molecules in the final models, |F_o_-F_c_| electron density difference maps, referred to as ‘omit maps’, were prepared by excluding ligands from F_c_ calculation, several cycles of refinment in *REFMAC5* and inspection of the maps in *COOT*. The validation tools in *COOT* and the *MolProbity* server [42] were used to assess model geometry. Comparisons with structures in the PDB were performed using the *DALI* server [28]. The *Protein Interfaces, Surfaces & Assemblies* server, [43] was used to calculate surface and interface areas and figures were prepared with *PyMOL*[44].

### Sequence analyses

A search in UniProt for IspF sequences using the E.C. number [4.6.1.12] returned 2458 entries. The list was filtered down to 2229 by excluding the bifunctional IspDF examples and fragmentary protein sequences. Sequences with greater than or equal to 90% identity were clustered and a single representative sequence identified for each cluster, reducing the list to 876 sequences. These were inspected and a decision taken to remove a number of sequences that still appeared to be fragmentary. A final set of 854 IspF sequences were submitted to the European Bioinformatics Institute-*ClustalOmega* multiple sequence alignment server [45]. The alignment was displayed in *Jalview*[46] and percentage conservation scores were manually calculated for all residues marked in Figure 3.

## Abbreviations

CDP: Cytidine 5′-diphosphate; CDP-MEP: 4-diphosphocytidyl-2*C*-methyl-D-erythritol-2-phosphate; CMP: Cytidine 5′-monophosphate; IspF: 2*C*-methyl-D-erythritol-2,4-cyclodiphosphate synthase; MEcPP: 2*C*-methyl-D-erythritol-2,4-cyclodiphosphate; MEP: 2*C*-methyl-D-erythritol-4-phosphate; MVA: Mevalonate; NCS: Non-crystallographic symmetry; RMSD: Root-mean-standard deviation; SDS-PAGE: Sodium dodecyl sulfate polyacrylamide gel electrophoresis.

## Competing interests

The authors declare they have no competing interests.

## Authors’ contributions

Conceived and designed the experiments: PEFO’R JK-T AD WNH. Performed the experiments: PEFO’R JK-T PKF AD. Analysed the data: PEFO’R JK-T PKF WNH. Wrote the paper: PEFO’R JK-T WNH. All authors read and approved the final manuscript.
